# Impact of obesity control on circulating level of endothelial progenitor cells and angiogenesis in response to ischemic stimulation

**DOI:** 10.1186/1479-5876-10-86

**Published:** 2012-07-11

**Authors:** Yung-Lung Chen, Chia-Lo Chang, Cheuk-Kwan Sun, Chiung-Jen Wu, Tzu-Hsien Tsai, Sheng-Ying Chung, Sarah Chua, Kuo-Ho Yeh, Steve Leu, Jiunn-Jye Sheu, Fan-Yen Lee, Chia-Hung Yen, Hon-Kan Yip

**Affiliations:** 1 Division of cardiology, Department of Internal Medicine, Kaohsiung Chang Gung Memorial Hospital and Chang Gung University College of Medicine, Kaohsiung, Taiwan; 2 Division of Colorectal Surgery, Department of Surgery, Kaohsiung Chang Gung Memorial Hospital and Chang Gung University College of Medicine, Kaohsiung, Taiwan; 3 Department of Emergency Medicine, E-Da Hospital, I-Shou University, Kaohsiung, Taiwan; 4 Center for Translational Research in Biomedical Sciences, Kaohsiung Chang Gung Memorial Hospital and Chang Gung University College of Medicine, Kaohsiung, Taiwan; 5 Division of Cardiovascular Surgery, Department of Surgery, Kaohsiung Chang Gung Memorial Hospital and Chang Gung University College of Medicine, Kaohsiung, Taiwan; 6 Department of Biological Science and Technology, National Pingtung University of Science and Technology, Pingtung, Taiwan

**Keywords:** Obesity control, Endothelial progenitor cells, Angiogenesis, Critical limb ischemia

## Abstract

**Background and aim:**

We tested the hypothesis that obesity reduced circulating number of endothelial progenitor cells (EPCs), angiogenic ability, and blood flow in ischemic tissue that could be reversed after obesity control.

**Methods:**

8-week-old C57BL/6J mice (n = 27) were equally divided into group 1 (fed with 22-week control diet), group 2 (22-week high fat diet), and group 3 (14-week high fat diet, followed by 8-week control diet). Critical limb ischemia (CLI) was induced at week 20 in groups 2 and 3. The animals were sacrificed at the end of 22 weeks.

**Results:**

Heart weight, body weight, abdominal fat weight, serum total cholesterol level, and fasting blood sugar were highest in group 2 (all p < 0.001). The numbers of circulating EPCs (C-kit/CD31+, Sca-1/KDR + and CXCR4/CD34+) were lower in groups 1 and 2 than in group 3 at 18 h after CLI induction (p < 0.03). The numbers of differentiated EPCs (C-kit/CD31+, CXCR4/CD34+ and CD133+) from adipose tissue after 14-day cultivation were also lowest in group 2 (p < 0.001). Protein expressions of VCAM-1, oxidative index, Smad3, and TGF-β were higher, whereas the Smad1/5 and BMP-2, mitochondrial cytochrome-C SDF-1α and CXCR4 were lower in group 2 than in groups 1 and 3 (all p < 0.02). Immunofluorescent staining of CD31+ and vWF + cells, the number of small vessel (<15 μm), and blood flow through Laser Doppler scanning of ischemic area were lower in group 2 compared to groups 1 and 3 on day 14 after CLI induction (all p < 0.001).

**Conclusion:**

Obesity suppressed abilities of angiogenesis and recovery from CLI that were reversed by obesity control.

## Background

The dramatic increase in the prevalence of obesity is mainly due to living in an environment characterized by calorie-rich foods and lack of physical activity, especially in Western countries [1]. Abundant data have demonstrated that obesity predisposes to a variety of low-grade chronic and systemic inflammatory diseases, including insulin resistance, type 2 diabetes, fatty liver diseases, osteoarthritis, atherosclerosis and its complications [2-7]. Epidemiologic studies have shown that obesity constitutes a major health threat because of its associated morbidity and mortality, especially those from cardiovascular diseases [8-10].

Studies have previously demonstrated that endothelial progenitor cells (EPCs), which are premature hematopoietic stem cells mobilized into systemic circulation from bone marrow, are capable of differentiating into mature endothelial cells for endothelial repair in blood vessels [11,12]. They have been reported to migrate to ischemic area in response to ischemia for angiogenesis/vasculogenesis, thereby enhancing recovery of ischemia-related organ dysfunction [11-15]. Not only have EPCs been found to have a positive therapeutic impact on ischemic organ dysfunction in both clinical [16] and experimental [17] settings, their circulating levels have also been successfully used as clinical markers of disease progression [18]. Studies have previously further demonstrated that decreased circulating number of EPCs is correlated with cumulative cardiovascular risk [13,19,20]. On the other hand, the impact of obesity, a risk factor for cardiovascular diseases of growing importance, on endothelial injury and dysfunction [21] as well as circulating number of endothelial progenitor cells (EPCs) [7,22] has not been fully investigated, especially in the setting of a well-controlled animal model study. Of particular significance in translational research is that whether obesity control has a positive impact on circulating number of EPCs, angiogenesis ability in response to ischemic insult, and tissue blood flow under ischemic condition [23].

By using a high fat diet-induced obesity model, the aim of this study was to test the hypotheses that, in obese mice: 1) Ischemic stimulation leads to a decrease in the number of circulating EPCs that can be reversed by obesity control; 2) Tissue ischemia is accompanied by impaired angiogenic capacity that is alleviated by improving obesity condition.

## Materials and methods

### Ethics

All animal experimental procedures were approved by the Institute of Animal Care and Use Committee at Kaohsiung Chang Gang Memorial Hospital and performed in accordance with the Guide for the Care and Use of Laboratory Animals (NIH publication No. 85-23, National Academy Press, Washington, DC, USA, revised 1996).

### Animal model of obesity

Eight-week-old male C57BL/6J mice (n = 24), weighing 22-24 gm, (Charles River Technology, BioLASCO Taiwan Co., Ltd., Taiwan), were fed with high-fat diet (45 Kcal% fat; Research Diets, Inc) to create the diet-induced obesity model for the purpose of this study. According to the literature [24] and the instructions from the company (Research Diets, Inc), successful obesity induction was defined as an increase in mouse body weight more than 35% after 13 weeks’ feeding with the diet. Our study showed that, by the end of 12 weeks of feeding with high fat diet, 75% mice fit the criteria of obesity.

These 18 obese mice were then equally divided into group 2 (continuously fed with high fat diet for further 10 weeks – obese group) and group 3 [continuously fed with high fat diet for further 2 weeks, followed by standard mouse chow (i.e. control diet) for 8 weeks – obesity control group]. Another group (group 1) of aged-matched C57BL/6J mice (n = 9) fed with control diet for the same duration (i.e., total 22-week control die), which was also purchased from the same company (Research Diets, Inc), served as untreated controls.

### Animal model of critical limb ischemia (CLI) for stimulating EPC mobilization into circulation

By the end of 20 weeks after obesity induction, mice in group 2 (obese), and group 3 (obesity control) were anesthetized by inhalation of 2.0% isoflurane. Mice in group 1wihtout receiving CLI procedure served as normal controls for comparing the molecular-cellular parameters and blood flow in CLI area. The mice were placed in a supine position on a warming pad at 37°C with the left hind limbs shaved. Under sterile conditions, the left femoral artery, small arterioles, and circumferential femoral artery were exposed and ligated over their proximal and distal portions before removal. Blood (0.3 mL in each mouse) was sampled for quantification of EPCs using flow cytometry at 18 h and day 14 after CLI induction before the animals were sacrificed. To elucidate the baseline level of circulating EPCs, blood sample (0.3 mL) was also collected 5 days prior to CLI procedure in each group of mice.

### Measurement of blood flow with laser Doppler

The detailed procedure has been described in our recent report [25]. Briefly, the mice in groups 1, 2 and 3 were anesthetized by inhalation of 2.0% isoflurane prior to CLI induction and at days 2 and 14 after CLI procedure prior to be sacrificed (n = 9 for each group). Blood flow was assessed in both inguinal areas and hind limbs by a Laser Doppler scanner (moorLDLS, Moor, Co. UK) with the animals in a supine position on a warming pad at 37°C. The ratio of blood flow in left hind limb (ischemic) to that on the right side (normal) was computed by the scanner. The mice were sacrificed and the quadriceps muscle was collected for Western blot analysis and immunofluorescent (IF) and immunohistochemical (IHC) studies.

### Flow cytometric quantification of endothelial progenitor cells

For blood sampling at different time points (i.e. prior to CLI and at 18 h and on day 14 after induction of CLI), cardiac puncture instead of the venous route was adopted for blood sampling using a 30# needle. A flow cytometic method for identification of EPCs derived from peripheral blood has been reported in our recent studies [17,18]. Briefly, the isolated MNCs from 0.3 cc blood (3.0 x 10^5^) were incubated for 30 minutes at 4 ^0^C in a dark room with monoclonal antibodies against phycoerythrin (PE)- conjugated kinase insert domain-conjugating receptor (KDR) (BD Biosciences), the phycoerythrin (PE)- -conjugated CD34 (BD Biosciences), the phycoerythrin (PE)-conjugated CD31 (BioLegend), fluorescein isothiocyanate (FITC)-conjugated CXCR4 (BD Biosciences), fluorescein isothiocyanate (FITC)-conjugated sca-1 (BD Biosciences) and fluorescein isothiocyanate (FITC)-conjugated c-kit (BD Biosciences) to determine the EPC surface markers of c-kit/CD31, sca-1/KDR, and CXCR4/CD34. The control ligand (IgG-PE conjugate) was used to detect any nonspecific association and define a threshold for glycoprotein binding. For analysis of KDR, the MNCs were further incubated with PE-conjugated anti-mouse antibody made in goat. After staining, the MNCs were fixed in 1% of paraformaldehyde. Quantitative two-colored flow cytometric analysis was performed using a fluorescence-activated cell sorter (Beckman Coulter FC500 flow cytometer), i.e., using a method of double staining. Each analysis included 8,000 cells per sample. The assays for the EPCs in each sample were performed in duplicate, with the mean level reported.

### Isolation of adipose-derived endothelial progenitor cells from mice

Following the CLI procedure, animals under inhalational anesthesia of 2.0% isoflurane by the end of 20 weeks after obesity induction, adipose tissue surrounding the epididymis was carefully dissected and excised. Then 200-300 μL of sterile saline was added to every 0.5 g of tissue to prevent dehydration. The tissue was cut into < 1 mm^3^ size pieces using a pair of sharp, sterile surgical scissors. Sterile saline (37˚C) was added to the homogenized adipose tissue in a ratio of 3:1 (saline: adipose tissue), followed by the addition of stock collagenase solution to a final concentration of 0.5 units/mL. The centrifuge tubes with the contents were placed and secured on a Thermaline shaker and incubated with constant agitation for 60 ± 15 min at 37˚C. After 40 minutes of incubation, the content was triturated with a 25 mL pipette for 2-3 minutes. The cells obtained were placed back to the rocker for incubation. The contents of the flask were transferred to 50 mL tubes after digestion, followed by centrifugation at 600 g, for 5 minutes at room temperature. The fat layer and saline supernatant from the tube were poured out gently in one smooth motion or removed using vacuum suction. The cell pellet thus obtained was resuspended in 40 mL saline and then centrifuged again at 600 g for 5 minutes at room temperature. After being resuspended again in 5 mL saline, the cell suspension was filtered through a 100 μm filter into a 50 mL conical tube to which 2 mL of saline was added to rinse the remaining cells through the filter. The flow-through was pipetted into a new 50 mL conical tube through a 40 μm filter. The tubes were centrifuged for a third time at 600 g for 5 minutes at room temperature. The cells were resuspended in saline. An aliquot of cell suspension was then taken for cell culture in M199 culture medium for two weeks. Flow cytometric analysis was then performed for quantification of EPCs using double staining (C-kit/CD31, Sca-1/KDR, CXCR4/CD34) and single stain for CD133+ EPC after 2-week cell culture.

### Isolation of mitochondria

The ischemic muscle was excised and washed with buffer A (100 mM Tris-HCl, 70 mM sucrose, 10 mM EDTA, and 210 mM mannitol, pH 7.4). Samples were minced finely in cold buffer A and incubated for 10 minutes. All samples were homogenized in an additional 3 mL of buffer A using a motor-driven grinder. The homogenate was centrifuged twice at 700 g for 10 minutes at 4°C. The supernatant was centrifuged again at 8,500 g for 15 min, and the pellets were washed with buffer B (10 mM Tris-HCl, 70 mM sucrose, 1 mM EDTA, and 230 mM mannitol, pH 7.4). The mitochondria-rich pellets were collected and stored at −70°C.

### Western blot analysis

Equal amounts (10-30 μg) of protein extracts from ischemic quadriceps of the animals were loaded and separated by SDS-PAGE using 12% acrylamide gradients. The membranes were incubated with monoclonal antibodies against intercellular adhesion molecule (ICAM)-1 (1:100, Abcam), CXCR4 (1: 1000, Abcam), stromal cell-derived growth factor (SDF)-1α (1: 1000, Cell Signaling), vascular endothelial growth factor (VEGF) (1: 1000, Abcam), phospho-Smad 3 (1:1000, Cell Signaling), transforming growth factor (TGF)-β (1:500, Abcam), phospho-Smad1/5 (1:1000, Cell Signaling), bone morphogenic protein (BMP)-2 (1:500, Abcam), and cytochrome c (Cyt c) (1: 2000, BD). Signals were detected with HRP-conjugated goat anti- mouse or goat anti-rabbit IgG. The Oxyblot Oxidized Protein Detection Kit was purchased from Chemicon (S7150) for oxyblot protein analysis. Proteins were transferred to nitrocellulose membranes which were then incubated in the primary antibody solution (anti-DNP 1: 150) for two hours, followed by incubation with second antibody solution (1:300) for one hour at room temperature. The washing procedure was repeated eight times within 40 minutes. Immunoreactive bands were visualized by enhanced chemiluminescence (ECL; Amersham Biosciences) which was then exposed to Biomax L film (Kodak). For quantification, ECL signals were digitized using Labwork software (UVP). For oxyblot protein analysis, a standard control was loaded on each gel.

### Immunofluorescent (IF) staining

IF staining was performed for the examination of CD31+ and von Willebrand factor (vWF) + cells, two endothelial cell markers, using respective primary antibodies based on our recent study [25]. Mouse control IgG (Abcam) and irrelevant monoclonal antibody against nuclear protein SC35 (Sigma) were used as the controls in the current study.

#### Vessel density in limb ischemic area

Immunohistochemical (IHC) staining of blood vessels was performed with α-SMA (1:400) as primary antibody at room temperature for 1 h, followed by washing with PBS thrice. Ten minutes after the addition of the anti-mouse-HRP conjugated secondary antibody, the tissue sections were washed with PBS thrice. Then 3,3′ diaminobenzidine (DAB) (0.7 gm/tablet) (Sigma) was added, followed by washing with PBS thrice after one minute. Finally, hematoxylin was added as a counter-stain for nuclei, followed by washing twice with PBS after one minute. Three sections of quadriceps were analyzed in each mouse for quantification of small vessel (≤ 15.0 μm) (200x) in ischemic region (for obese mice with and without obesity reduction) and non-ischemic quadriceps (for age-matched normal control). For quantification and statistical analysis, three randomly selected high-power fields [(HPFs) (200x)] were analyzed in each section under the microscope. The mean number ± SD of small vessels per HPF for each animal was then determined by summation of all numbers divided by 9 (i.e., 3 sections x 3 randomly selected HPFs = 9). The procedure and protocol for measurement of vessel density in limb ischemic region was based on our recent report [25].

### Statistical analysis

Quantitative data are expressed as means ± SD. Statistical analysis was adequately performed by ANOVA followed by Bonferroni’s multiple-comparisons post hoc test. Statistical analysis was performed using SAS statistical software for Windows version 8.2 (SAS institute, Cary, NC). A probability value <0.05 was considered statistically significant.

## Results

### The baseline relevant variables

The initial body weight and fasting blood sugar were similar among animals in group 1 (normal control), group 2 (obese) and group 3 (obesity induction followed by obesity control). However, the final body weight was significantly higher in group 2 than in groups 1 and 3, but it showed no difference between the later two groups (Table 1). Additionally, final fasting blood sugar, abdominal fat weight and serum cholesterol were remarkably higher in group 2 than in groups 1 and 2, and significantly higher in group 3 than in group 1.

**Table 1:** Baseline Characteristics and Flow Cytometry for EPCs

Variables	Control (n = 9)	Obesity (n = 9)	Obesity-C (n = 9)	P* value
Initial body weight (g)	26.3 ± 0.70	25.2 ± 0.61	25.2 ± 0.66	0.655
Final body weight (g)	34.9 ± 1.42^a^	43.7 ± 1.50^b^	35.6 ± 2.19^a^	<0.0001
Final abdominal fat weight (g)	1.59 ± 0.45^a^	4.14 ± 0.69^b^	2.37 ± 0.14^c^	<0.0001
Final total cholesterol (mg/dl)	138.1 ± 10.2^a^	231.2 ± 7.59^b^	189.8 ± 7.55^c^	<0.001
Initial blood glucose (mg/dl)†	98.8 ± 9.4	102 ± 8.2	100.3 ± 8.6	0.667
Final blood glucose (mg/dl)†	144.2 ± 17.4^a^	306.9 ± 71.4^b^	182.2 ± 18.8^c^	<0.0001
C-kit/CD31 (%)				
Baseline	1.06 ± 0.40	1.15 ± 0.21	1.21 ± 0.37	0.555
18 hour after CLI procedure	1.20 ± 0.44^a^	2.60 ± 0.49^b^	4.11 ± 0.98^c^	<0.0001
Day 14 after CLI procedure	1.02 ± 1.01^a^	0.75 ± 0.23^a^	2.40 ± 0.82^b^	0.0019
Sca-1/KDR (%)				
Baseline	0.66 ± 0.21	0.85 ± 0.27	0.73 ± 0.39	0.461
18 hour after CLI procedure	0.87 ± 0.25^a^	1.15 ± 0.24^a^	1.88 ± 0.16^b^	0.006
Day 14 after CLI procedure	0.56 ± 0.29	0.38 ± 0.20	0.89 ± 0.56	0.124
CXCR4/CD34 (%)				
Baseline	0.43 ± 0.27	0.38 ± 0.17	0.56 ± 0.45	0.789
18 hour after CLI procedure	0.54 ± 0.34^a^	0.68 ± 0.31^a^	1.34 ± 0.34^b^	0.006
Day 14 after CLI procedure	0.63 ± 0.69	0.37 ± 0.19	0.79 ± 0.70	0.764

*: by one-way ANOVA on log transformed data.

Different letters (^a, b, c^) associated with different groups indicate significant difference (at 0.05 level) by Tukey multiple comparison procedure.

† value was observed after 12 fasting in the mice.

C = control; CLI = critical limb ischemia.

### Serial changes of circulating EPCs in three groups of animals after CLI

The circulating numbers of EPCs, including C-kit/CD31+, Sca-1/KDR+, CXCR4/CD34+ cells, did not differ among animals in three groups prior to CLI induction. However, the circulating numbers of these EPCs were significantly higher in group 3 than in groups 1 and 2 by 18 h after CLI procedure (Table 1). Additionally, the circulating number of C-kit/CD31+ EPC was notably higher in group 2 than in group 1. However, other two circulating numbers of Sca-1/KDR+, CXCR4/CD34+ EPCs did not differ between groups 1 and 2 at 18 h following the CLI procedure.

By day 14 after CLI induction, the circulating number of C-kit/CD31+ EPC was still notably higher in group 3 than in groups 1 and 2, but it showed no difference between latter two groups. On the other hand, those circulating numbers of Sca-1/KDR + and CXCR4/CD34+ EPCs were similar among the three groups by 14 after CLI procedure (Table 1).

### Adipose-derived endothelial progenitor cells (ADEPCs)

**Figure 1:**
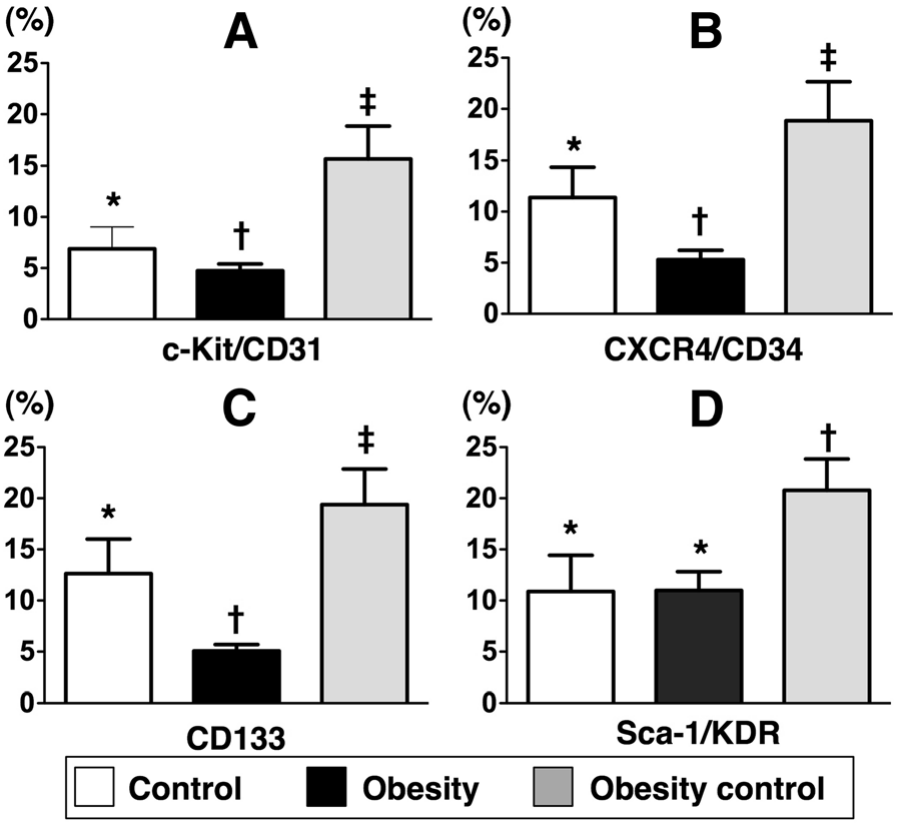
**Differentiation of adipose derived endothelial progenitor cells (ADEPCs).A** to **D)** Showing numbers of C-kit/CD31+, CXCR4/CD34+, CD133+ and Sca-1/KDR + EPCs after 14-day cell culturing, respectively. * vs. other groups with different symbols (i.e., * vs. † vs. ‡), p < 0.001. Statistical analysis by ANOVA followed by Bonferroni multiple comparison post hoc test (n = 9 in each group).

To determine whether weight reduction would affect EPC differentiation in fat tissue, adipose-derived cells were cultured in M199 culture medium (i.e., endothelial cell culture medium) for 14 days, followed by flow cytometric analysis. Interestingly, the numbers of ADEPCs, including C-kit/CD31+, CXCR4/CD34+ (double staining) and CD133+ (i.e., single stain), were remarkably higher in group 3 than in groups 1 and 2, and significantly higher in group 1 than in group 2 (Figure 1). On the other hand, the number of Sca-1/KDR + ADEPC was similar between groups 1 and 2. However, this biomarker was significantly higher in group 3 than in groups 1 and 2.

### Protein expressions of fibrotic and anti-fibrotic biomarkers in ischemic tissue

**Figure 2:**
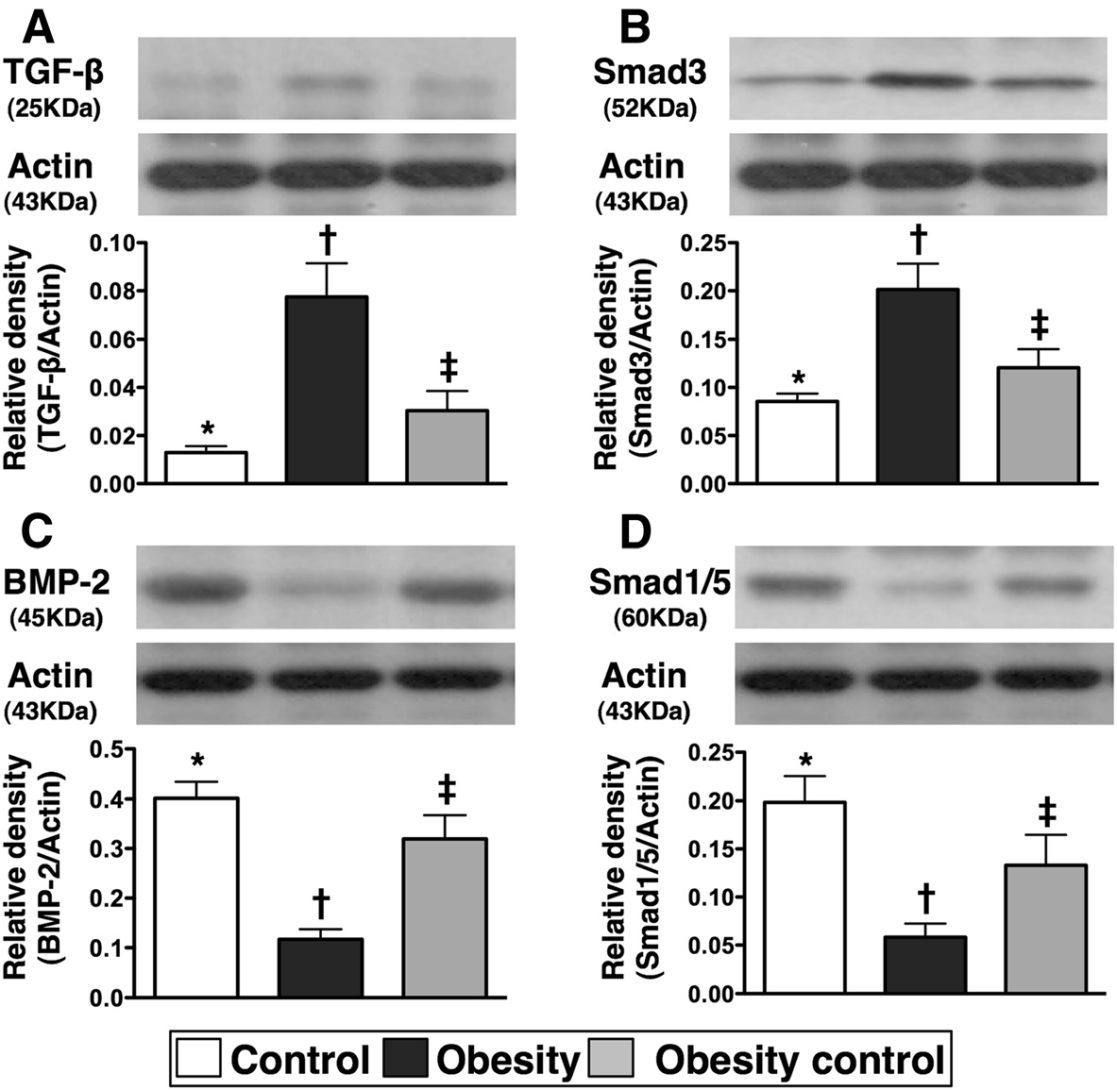
**Western blot results of fibrotic and anti-fibrotic biomarkers in ischemic muscle by day 14 after critical limb ischemia (CLI) procedure (n = 9). A & B)** protein expressions of transforming growth factor (TGF)-β and phosporylated-Smad3. * vs. other groups with different symbols, p < 0.002. **C & D)** protein expressions of bone morphogenic protein (BMP)-2 and phosporylated-Smad1/5. * vs. other groups with different symbols, p < 0.003. Statistical analysis by ANOVA followed by Bonferroni multiple comparison post hoc test.

The protein expressions of TGF-β and phosphorylated-Smad 3, two indexes of fibrosis in ischemic muscle, were notably higher in group 2 than in groups 1 and 2, and they were significantly higher in group 3 than in group 1 (Figure 2). By contrast, the protein expressions of BPM-2 and phosphorylated-Smad1/5, two anti-fibrotic biomarkers in ischemic muscle, were significantly lower in group 2 than in groups 1 and 3, and notably lower in group 3 compared to group 1.

### Cytochrome C protein expression in mitochondria and cytosol in ischemic tissue

**Figure 3:**
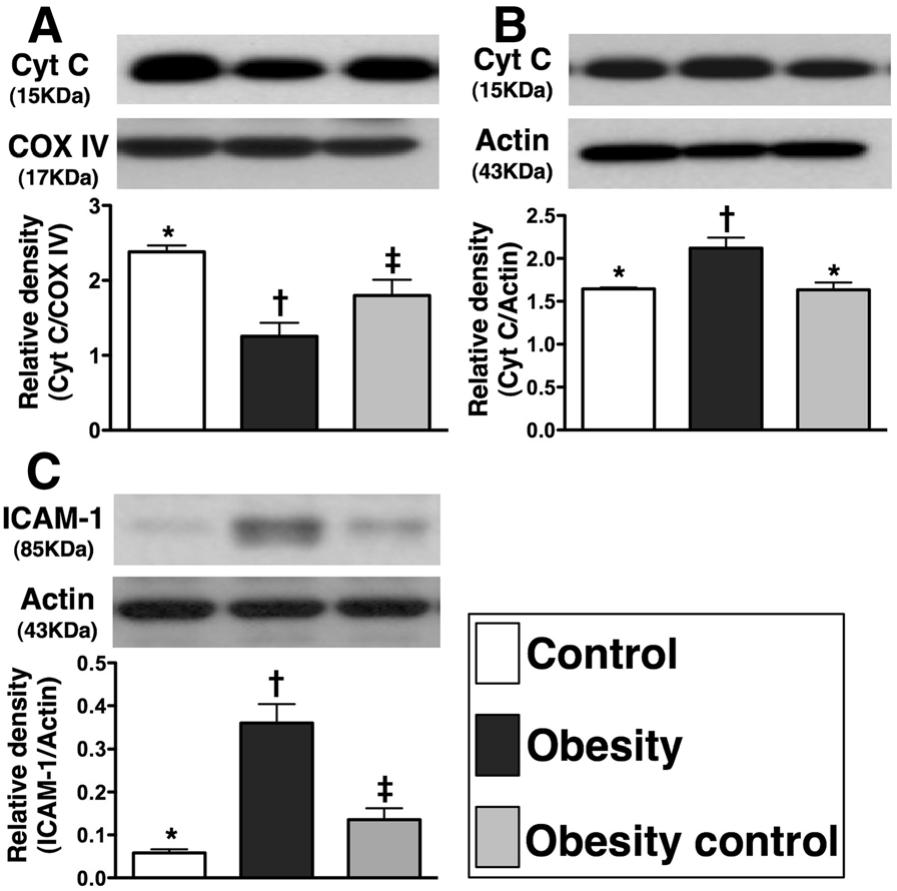
**Western blot results of mitochondrial-storage and inflammatory biomarkers by day 14 following CLI procedure (n = 9). A)** protein expression of mitochondrial cytochrome C (Cyt C) in CLI area. * vs. other groups with different symbols, p < 0.03. **B)** protein expression of cytosolic Cyt C in CLI area. * vs. other groups with different symbols, p < 0.003. **C)** Total protein expression of intercellular adhesion molecule (ICAM)-1 in CLI area. * vs. other groups with different symbols, p < 0.001. Statistical analysis by ANOVA followed by Bonferroni multiple comparison post hoc test.

The total mitochondrial cytochrome C protein expression (Figure 3-A) was significantly lower in group 2 than that in groups 1 and 3, and notably lower in group 3 than in group 1. Conversely, total amount of cytosolic cytochrome C protein expression (Figure 3-B) was significantly higher in group 2 than that in groups 1 and 3, but no significant difference was noted between groups 1 and 3. These findings indicate that the expression of cytochrome C, an index of energy supply and storage in mitochondria, was notably lower in group 2 than in groups 1 and 3. Besides, the increase in cytosolic cytochrome C content in group 2 also suggest significant mitochondrial damage with cytochrome C release into the cytosol in the ischemic muscle. These findings imply that weight reduction significantly alleviated the intensity of oxidative stress resulting from free radical production via a change in permeability of the mitochondrial transition pore.

### The protein expressions of inflammatory, oxidative stress biomarkers and angiogenesis factors in ischemic tissue

**Figure 4:**
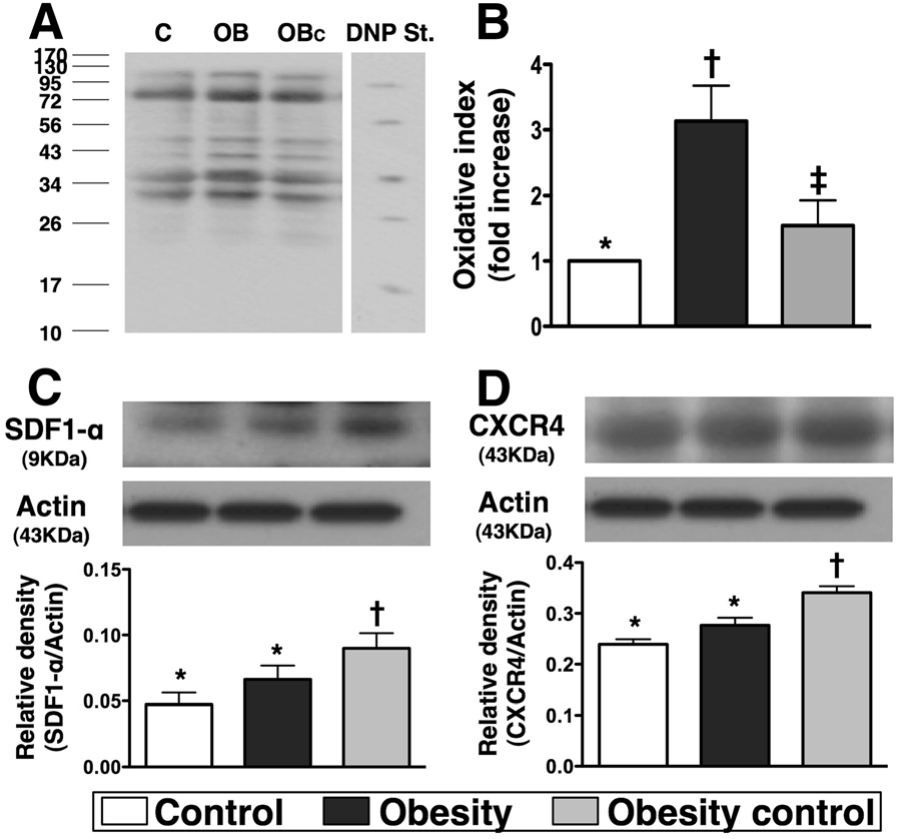
**Western blot results of oxidative stress and angiogenic factors by day 14 after CLI procedure (n = 9). A & B)** Significantly higher oxidative index, protein carbonyls, in obesity animals than in normal and obesity controls, and significantly higher in obesity-control animals than in normal controls. * vs. other groups with different symbols, p < 0.005. (Note: Right lane and left lane shown on upper panel represent control oxidized molecular protein standard and protein molecular weight marker, respectively). DNP = 1-3 dinitrophenylhydrazone. **C & D)** protein expressions of stromal cell-derived factor (SDF)-1α and CXCR4 were significantly enhanced in obesity-control mice than in obesity mice in response to ischemic stress in ischemic muscle. * vs. other groups with different symbols, p < 0.02. Statistical analysis by ANOVA followed by Bonferroni multiple comparison post hoc test.

The protein expression of ICAM-1 (Figure 3-C), an indicator of inflammation, was markedly increased in group 2 than in groups 1 and 3, and significantly increased in group 3 than in group 1 by day 14 after the CLI procedure. Additionally, Western blot analysis (Figure 4-A &4-B) showed that mitochondrial oxidative stress was significantly higher in group 2 than that in groups 1 and 3, and notably higher in group 3 than in group 1.

The protein expressions of SDF-1α (Figure 4-C) and CXCR4 (Figure 4-D), two indicators of angiogenesis in muscle of ischemic area in response to ischemic stress, were significantly increased in group 3 than in groups 1 and 2, but there was no significant difference between groups 1 and 2. These findings suggest that group 3 animals had enhanced SDF-1α expression in ischemic tissue in response to ischemic stimulation for attracting the homing of CXCR4+ cells into the ischemic region to participate in angiogenesis.

### Immunofluorescent (IF) and immunohistochemical (IHC) staining in ischemic tissue

**Figure 5:**
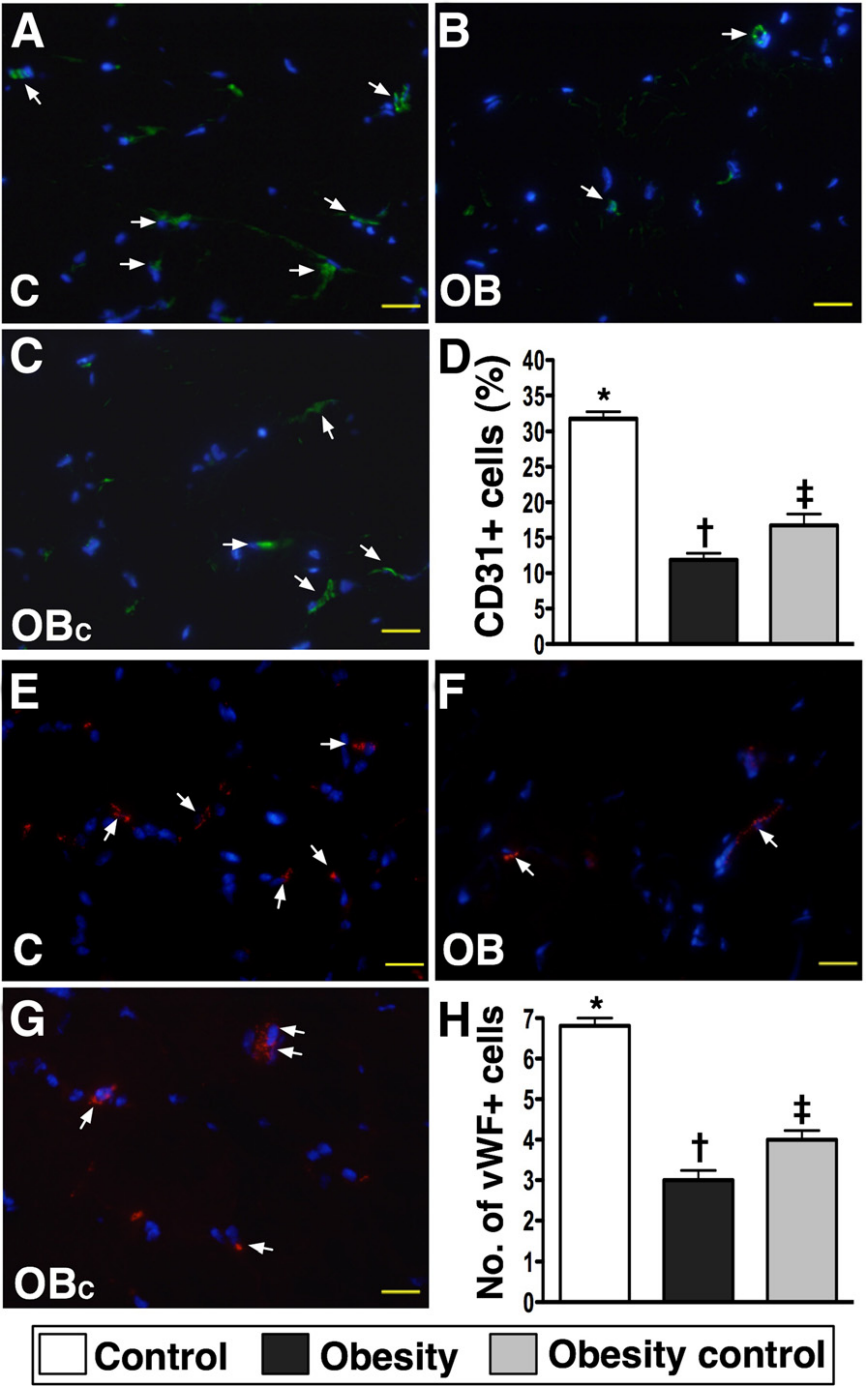
**Quantifications of endothelial cells (ECs) in ischemic region by day 14 after CLI procedure using immunofluorescent microscope (400x) (n = 9). A to C)** showing the numbers of CD31+ ECs (white arrows) remarkably higher in normal control (C) group than in obesity (OB) and obesity control (OB_C_) groups, and significantly higher in OB_C_ group than in OB group. **D)** * vs. other groups with different symbols, p < 0.0001. **E to G)** showing the numbers of von Willebrand factor (vWF) + ECs (white arrows) notably higher in C group than in OB and OB_C_ groups, and significantly higher in OB_C_ group than in OB group. **G)** * vs. other groups with different symbols, p < 0.0001. Statistical analysis by ANOVA followed by Bonferroni multiple comparison post hoc test. The scale bars in right lower corner represent 20 μm.

IF staining showed that the numbers of CD31+ and vWF + cells, two indicators of endothelial cell markers, were significantly lower in group 2 than in groups 1 and 3, and notably lower in group 3 than in group 1 on day 14 in ischemic tissue after the CLI procedure (Figure 5).

**Figure 6:**
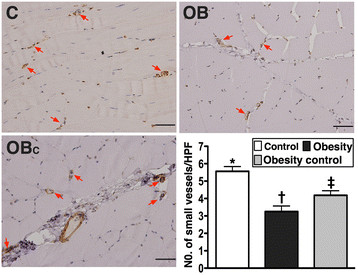
**Immunohistochemical staining of alpha-smooth muscle actin for quantification of small vessel (≤ 15.0 μm) (200x) in ischemic quadriceps at day 14 after CLI induction (n = 9). A to D)** Significantly higher number of small vessels (≤ 15 μm) (red arrows) in normal control (C) group than in obesity (OB) and obesity control (OB_C_) groups, and notably higher number in OB_C_ group than in OB group. * vs. other groups with different symbols, p < 0.01. Statistical analysis by ANOVA followed by Bonferroni multiple comparison post hoc test. Scale bars in right lower corner represent 50 μm. HPF = high-power field (200x).

IHC staining revealed remarkably lower number of small vessels (≤ 15 μm in diameter) in ischemic muscle in group 2 than that in groups 1 and 3, and it was significantly lower in group 3 compared with that in group 1 on day 14 following CLI induction (Figure 6).

### Laser Doppler analysis of blood flow

**Figure 7:**
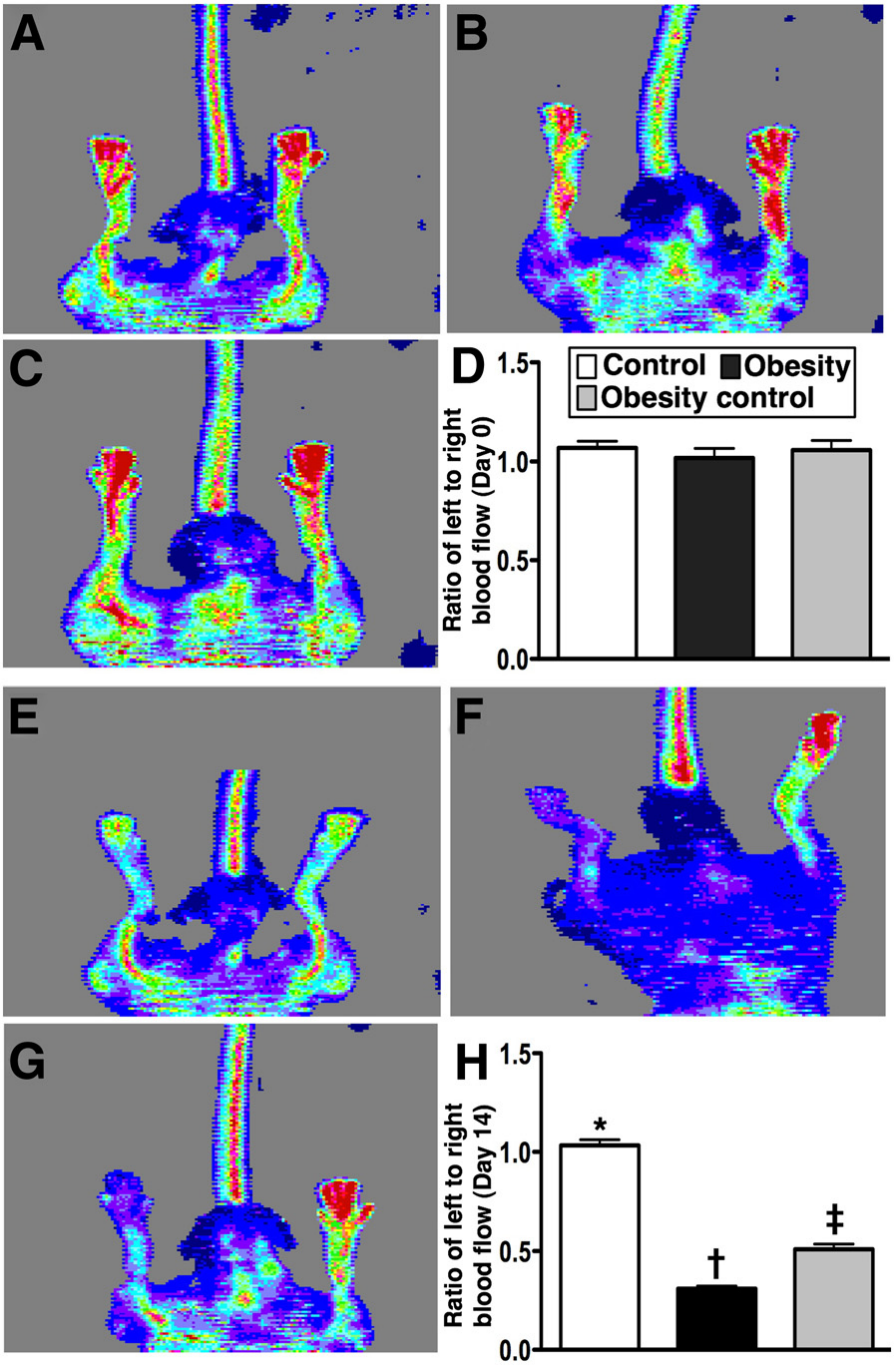
**Laser Doppler scanning of hind limb blood flow on day 14 after CLI induction (n = 9). A to C & D)** Normal blood flow in both hind limbs in normal control (A), obesity (B) and obesity control (C) prior to the procedure. **E to G)** Significantly reduced ratio of ischemic/normal blood flow (INBF) in obesity (F) group than in obesity control (G) and normal control (E) groups, and notably lower in obesity control group than in normal controls by day 14 after CLI procedure. **H)** * vs. other groups with different symbols, p < 0.001. Statistical analysis by ANOVA followed by Bonferroni multiple comparison post hoc test.

The ratio of ischemic/normal blood flow (INBF) did not differ among groups 1, 2, and 3 prior to CLI induction. There was also no significant difference between groups 2 and 3, but it was markedly reduced in these two groups of animals compared with that in group 1 on day 2 after the CLI procedure (Figure 7). By day 14 after CLI induction, the ratio of INBF was significantly reduced in group 2 than that in groups 1 and 3, and significantly lower in group 3 than in group 1.

## Discussion

The present study, which investigated the impact of obesity control on the differentiation of ADEPCs, circulating level of EPCs, and angiogenesis in response to ischemic stimulation, yields several striking implications. First, obesity suppressed the ability of ADEPC differentiation that was reversed by obesity control. Second, the circulating level of EPCs in response to ischemia was notably reduced in obesity as compared with obesity control. Third, the angiogenic capacity in response to ischemic stimulation was markedly impaired in the obese mice than in obesity control. Finally, the blood flow in ischemic muscle was remarkably decreased in obese animals but was significantly restored after obesity control.

### Impact of obesity control on circulating level of EPCs in response to ischemic stimulation

Obesity, which is a rapidly growing epidemic worldwide, is commonly associated with a broad range of cardiovascular diseases [1-8]. Although the association between increased cardiovascular risk and decreased circulating number of EPCs has been demonstrated in previous studies [13,19,20], the impact of obesity on endothelial injury and dysfunction [21] and circulating number of EPCs [7,22] has not been fully examined. Besides, the effect of obesity control (i.e., body weight reduction) on vascular integrity has seldom been explored [22], especially in a well-controlled study using animal model. One important finding in the current study is that, although the circulating numbers of EPCs (C-kit/CD31+, Sca-1/KDR+, CXCR4/CD34+) showed no significant difference among the control, obese, and obesity control animals before CLI induction (Table 1), they were remarkably reduced in obese mice at the acute stage (i.e. 18 h after the CLI procedure) compared with that in the obesity controls. Additionally, the circulating number of C-kit/CD31+ cells remained notably higher in the obesity-controlled mice than that in the obese animals at the recovery stage (i.e., at day 14 after CLI). These findings, in addition to implicating a poorer angiogenic response to ischemic stress in the obese mice, also support the beneficial effects of weight reduction from previous clinical observation studies [7,22].

### Beneficial effect of obesity control on enhancing the differentiation of adipose-derived endothelial progenitor cells (ADEPCs)

Growing data have shown distinct advantages of using adipose-derived stem cells in improving ischemia-related organ dysfunction [26,27]. However, whether obesity also influences the differentiation of ADEMPC is currently unclear. Another important finding in the present study is that the number of differentiated EPCs (C-kit+, CXCR4/CD34+, CD133+) from adipose tissue after 14-day cell culturing was significantly lower in the obese mice compared with that in the normal controls. The number, on the other hand, was restored to the level of the normal controls in obesity-controlled mice. Interestingly, a previous study [23] has demonstrated that the colony-forming capacity of peripheral blood-derived EPCs was notably impaired in overweight and obese adults. Accordingly, our findings not only are comparable to those of a previous study [23], but also highlight the possible therapeutic potential of obesity control in improving the capacity of ADEPC differentiation, especially in obese patients who are candidates for ADPEC therapy due to ischemia-related organ dysfunction in our future clinical practice.

### Role of obesity reduction in attenuating inflammation, oxidative stress, fibrosis, apoptosis, and mitochondrial damage in ischemic tissue

The links among chronic inflammation, increased oxidative stress, and cardiovascular diseases have been well documented [28-31]. Additionally, apoptosis, fibrosis, and degree of mitochondrial damage have been clearly identified as useful biomarkers for predictive of outcome in experimental studies of cardiovascular diseases [17,25-28]. However, the relationship between obesity and these biomarkers in the setting of organ ischemia remains uncertain. An essential finding in the current study is that the inflammatory, oxidative stress, fibrotic, and mitochondrial damage biomarkers (Figures 2, 3 and 4) were substantially increased, whereas the anti-fibrotic biomarkers were remarkably reduced in ischemic muscle of obese animals compared to those in control animals. These findings suggest that obesity may participate in the up-regulation of these cardiovascular disease-related biomarkers after an acute ischemic insult. Of particular importance is that these inflammatory, oxidative stress, fibrotic, and mitochondria damage biomarkers were markedly decreased, whereas the anti-fibrotic biomarkers were notably increased in the obese mice after obesity control (Figures 2, 3 and 4). Our findings, therefore, further support the benefit of obesity control in ameliorating the risk for cardiovascular diseases.

### Probability of obesity control in improving angiogenesis and blood flow in ischemic tissue

The principal finding in the present study is that the protein expressions of CXCR4 and SDF-1α, two importantly angiogenic factors in response to ischemic stimulation (Figure 4), were notably impaired in obese animals. Additionally, as compared with the normal controls, other angiogenic biomarkers, including immunofluorescent staining (CD31+ and vWF + cells) (Figure 5) and neovascularization (i.e., number of small vessels) (Figure 6) were significantly lower in the ischemic muscle of the obese animals by day 14 after the CLI procedure. These parameters, on the other hand, were significantly preserved after obesity control. Importantly, the blood flow in ischemic muscle (Figure 7) was notably restored in obese animals after obesity control compared to those without. These findings again suggest a positive therapeutic impact of weight reduction on recovery after an ischemic insult.

### Study limitations

**Figure 8:**
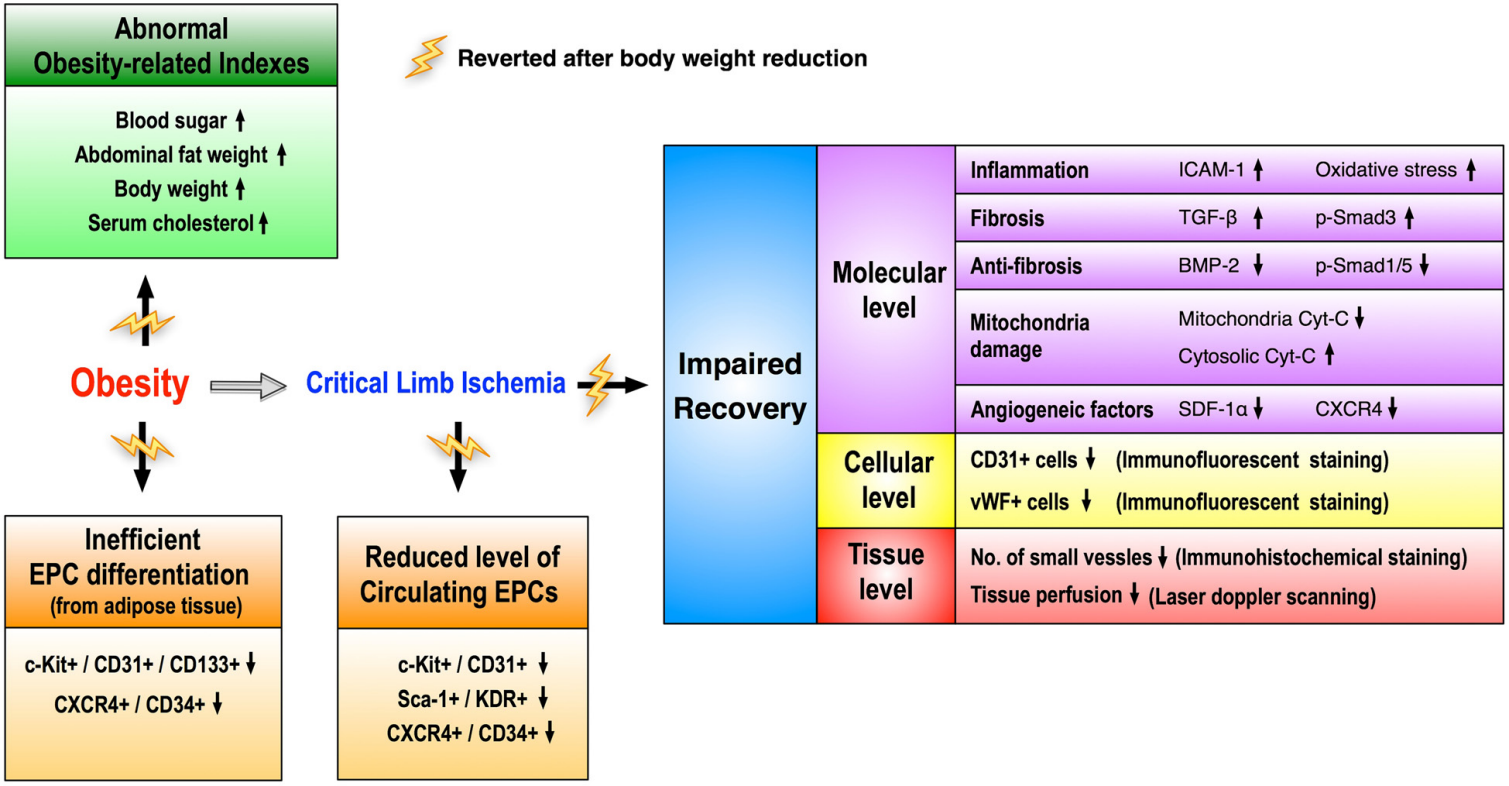
**Proposed mechanisms underlying the effects of obesity control (i.e., body weight reduction) on CLI in a murine model based on the findings of the present study.** BMP-2 = bone morphogenic protein 2; Cyt-C = cytochrome C; EPC = endothelial progenitor cells; ICAM-1 = intercellular adhesion molecule 1; SDF-1α = stromal cell-derived factor-1 alpha; TGF-β = transforming growth factor beta.

This study has limitations. First, the exact mechanisms underlying the partial restoration of angiogenic capacity after control of obesity in this experimental setting of tissue ischemia remain unclear. The proposed mechanisms, which are based on our findings, have been summarized in Figure 8. Second, the lack of significant difference in the baseline level of circulating EPCs among the three groups of animals was unexpected. It could be due to the possibility that the duration of obesity induction was too short to initiate overt cardiovascular damage in our experimental setting. Third, the reason for the elevated number of a specific population of ADEPCs (i.e., C-kit/CD31+, Sca-1/KDR+) in the obesity-controlled mice compared to that in the normal controls after 14-day cell culture is still unclear. It may be due to a hyper-reactive response to ischemic stimulation that occurs only at the early stage of weight reduction.

## Conclusion

Obesity impaired elevation of circulating level of EPCs in response to ischemic stimulation, hampered the differentiation of EPCs from adipose tissue, and impeded the capacity of angiogenesis and blood flow in ischemic tissue that were significantly restored by obesity control in a murine model of diet-induced obesity with limb ischemia.

## Misc

Chia-Lo Chang and Yung-Lung Chen equal contributors.

## Competing interests

The authors declare that they have no competing interests.

## Authors’ contributions

All authors have read and approved the final manuscript. YLC, CLC, CKS, and CJW designed the experiment, drafted and performed animal experiments. THT, SYC, SC, KHY, SL, JJS, FYL, and CHY were responsible for the laboratory assay and troubleshooting. YLC, CLC, and HKY participated in refinement of experiment protocol and coordination and helped in drafting the manuscript. All authors report no disclosures and have any commercial associations or interests, including consultancies, stock ownership or other competing equity interest.
